# Multi-Collaborator Engagement to Identify Research Priorities for Early Intervention in Cerebral Palsy

**DOI:** 10.3390/jcm14217592

**Published:** 2025-10-26

**Authors:** Angela Shierk, Nancy J. Clegg, Daralyn Fulton, Mauricio R. Delgado, Vanessa Hunt, Janet Bettger, Sydney Chapa, Sadie Oakley, Heather Roberts

**Affiliations:** 1 Department of Clinical Research, Scottish Rite for Children, Dallas, TX 75219, USA; 2 Department of Applied Clinical Research, UT Southwestern Medical Center, Dallas, TX 75390, USA; 3 Department of Neurology, UT Southwestern Medical Center, Dallas, TX 75390, USA; 4 Department of Occupational Therapy, Texas Woman’s University, Denton, TX 76204, USA; 5 Department of Physical Medicine and Rehabilitation, The University of North Carolina at Chapel Hill School of Medicine, Chapel Hill, NC 27599, USA

**Keywords:** cerebral palsy, early diagnosis, early intervention, research priorities, patient-centered outcomes research

## Abstract

**Background/Objectives**: Although clinical practice guidelines and evidence-based practices for early cerebral palsy (CP) diagnosis and treatment are well established, their implementation remains inconsistent across care settings. This study sought to identify key research priorities related to early CP diagnosis and treatment and to develop an actionable framework through multi-collaborator consensus building. **Methods**: The 97 adult participants included 42 who have lived experience with CP. Before the conference, participants completed a survey rating the importance of research topics. During the conference, aggregated results were presented, followed by 16 focus group discussions to refine research priorities. A follow-up survey was conducted to validate the final priorities. **Results**: Six actionable items were identified: improving diagnosis communication, ensuring early referrals and interdisciplinary collaboration, creating inclusive education and training, scaling evidence-based therapies and researching new interventions, developing social support systems, and advocating for policy and cultural change. A research framework was developed that outlines how these priorities can be addressed through three main strategies: education and training, research expansion, and policy advocacy. **Conclusions**: This study highlights the critical need for comprehensive and compassionate care for families receiving a CP diagnosis. Key priorities include early detection, coordinated multidisciplinary teams, and well-trained professionals delivering evidence-based interventions. The comprehensive framework addressing these priorities lays the foundation for future patient-centered comparative clinical effectiveness research.

## 1. Introduction

Cerebral palsy (CP) includes a group of permanent motor disorders caused by non-progressive disturbances in the developing fetal or infant brain [1]. CP has a heterogeneous etiology, leading to varied impairments and functional presentations, but the primary characteristic is lifelong difficulty with posture and movement, affecting gross and fine motor skills. More than 10,000 children in the U.S. are born with CP each year, making it the most common childhood physical disability [2]. Individuals with CP commonly have comorbidities, including epilepsy, musculoskeletal problems, sensory impairments, and cognitive and communication difficulties. Therefore, many children with CP require a multidisciplinary care team, requiring significant time and effort from families to access and coordinate services. Moreover, access to this coordinated care hinges on timely diagnosis, a current challenge in CP treatment.

Historically, the average age of diagnosis was 12 to 24 months due to a “wait and see” approach. However, in 2017, an international clinical practice guideline presented evidence that diagnosis can be made accurately before a child is 6 months of age [3]. Early diagnosis allows children to access critical early interventions, defined as services implemented between birth and age 3 years. Neural circuits for learning, behavior, and health are most adaptable in the first 2 years of life [4]. High-quality early intervention can improve developmental outcomes, enhance family and community well-being, and positively influence school readiness and lifelong learning [5].

Despite the availability of evidence-based early detection guidelines for CP, significant gaps in implementation persist [6], delaying access to CP-specific interventions during critical developmental windows. Studies in multiple parts of the world have identified substantial barriers to early identification and diagnosis of CP by healthcare and education providers particularly lack of training in recommended early detection tools compounded by systemic challenges including insufficient time, heavy workloads, staffing shortages, limited funding for professional development, and restrictive organizational policies [7,8,9,10].

While these findings illuminate global challenges, it remained unclear whether similar barriers existed in the southwestern United States or what research priorities would best address the needs of young children with CP in our region. Local verification was critical to developing context-specific strategies for strengthening guideline implementation, building provider capacity, fostering interdisciplinary collaboration, and addressing the unique structural and policy factors affecting timely diagnosis and intervention in our community. In response, we established a CP Task Force, a multi-collaborator group, to engage and train individuals in patient-centered comparative clinical effectiveness research (CER) methodologies. As part of that effort, the CP Task Force hosted a conference to establish a CER agenda focused on comparing standard-of-care interventions and processes to understand which interventions and processes work best for which young children with CP and their families.

However, because of inconsistent implementation of early detection guidelines and evidence-based interventions in our region, a fragmented care landscape existed where “standard care” was poorly defined. When we attempted to develop CER questions, we discovered that the variability in standard care resulted in questions that often focused on comparing evidence-based practices to outdated or suboptimal care. This revealed that we first needed to characterize current care practices, understand patient and family experiences and research priorities, and identify implementation barriers, all essential groundwork to enable meaningful CER questions. The aim of this paper is to present the overarching research priorities and framework that emerged from this foundational work.

## 2. Materials and Methods

The study team conducted a mixed-methods study comprising surveys, focus groups, and data integration to identify research priorities for young children with CP and their families. The study protocol was approved by the University of Texas Southwestern Medical Center’s Institutional Review Board (IRB) (STU-2024-0022) on 20 March 2024. The IRB waived the need for signed consent for the collection, analysis, and publication of anonymized data for this non-interventional study. The research team, the CP Task Force, included physicians, occupational therapists, physical therapists, graduate students, nurses, researchers, therapeutic recreation specialists, caregivers, and adults with CP. With direct experience in CP care and advocacy, team members brought diverse perspectives that shaped study design, data collection, and interpretation, enhancing credibility and relevance. Reflexive discussions throughout the process helped mitigate bias and ensure a holistic, inclusive approach.

### 2.1. Preconference Survey

To develop a quantitative pre-conference survey, we searched PubMed, Google Scholar, and CINAHL in May and June 2024, using the terms ‘cerebral palsy’ and ‘research priorities,’ and screened references of relevant articles for additional articles. From the 11 papers identified [3,11,12,13,14,15,16,17,18,19,20,21,22] we selected various research topics related to CP in young children. In August and September 2024, we offered the survey to clinicians, researchers, and family members of children with CP who had registered for the conference. Ratings were requested on a scale from 1 (“not important”) to 5 (“very important”), focusing on four key topics: early detection and diagnosis, collaboration for early treatment, timing and types of intervention, and the impact of early treatment on parents and parent education (Supplementary Material S1).

### 2.2. Conference Focus Groups

Conference attendees were divided into eight focus groups, each including individuals with lived experience (parents and people with CP), professionals (clinicians and researchers), and trainees (healthcare students) to foster collaborative dialogue. Each focus group met twice. A discussion guide was used to facilitate focus groups (Supplementary Material S2). In the first round, the participants discussed early detection/diagnosis and initiation of early intervention; in the second, they focused on early treatment and community resources. Mimicking the process of Novak et al., [23] participants noted practices/experiences they wanted to stop (red light), re-evaluate or improve (yellow light), or continue (green light), and what they hoped for (purple).

### 2.3. Post-Conference Validation Survey

Approximately one month following the conference, conference attendees were invited to complete a follow-up survey to validate the research priorities. The survey presented the overarching actionable items that were created based on the integration of data from the pre-conference survey and focus groups. Participants were asked to confirm whether these priorities aligned with discussions held during the conference, suggest modifications or additions, and reflect on how their participation influenced their perspectives on CP, early detection and intervention, patient-centered outcomes research, and advocacy. The survey also assessed participants’ likelihood of recommending the conference to colleagues using a 10-point scale (Supplementary Material S3).

### 2.4. Data Analysis

#### 2.4.1. Pre-Conference Survey

Mean ratings were calculated for each specific question within the four areas: early detection and diagnosis, collaboration for early treatment, timing and types of intervention, and the impact of early treatment on parents and parent education. These means identified which specific research topics were rated as most important within each area. Additionally, frequency counts were tabulated to determine how many participants selected each of the four overall topic areas as their single most important priority and how many selected it as their least important priority.

#### 2.4.2. Focus Groups

Audio recordings of the focus groups were transcribed verbatim and analyzed using a qualitative descriptive approach and thematic analysis [24,25]. Two independent reviewers coded the transcripts and written responses, with a third reviewer synthesizing themes by merging similar content. An adult with CP served as one of the primary reviewers of the focus group analysis. To enhance rigor, data from three sources, qualitative themes from the focus groups, notes made during the conference, and visual sketches created by a contracted observer were analyzed by two independent reviewers for triangulation. Any discrepancies were resolved through discussion among the three reviewers.

#### 2.4.3. Data Integration and Validation

Following Krefting’s recommendations for ensuring trustworthiness [26], data integration and validation occurred in multiple stages. First, qualitative themes from the focus groups were integrated with findings from the pre-conference survey to identify research priorities that emerged as most important across both data sources. These integrated findings were then rephrased into actionable items with concrete recommendations based on the data. The first stage of member checking involved sending these actionable items to conference participants through a conference follow-up survey to validate that the identified priorities accurately reflected conference attendees’ perspectives. Following validation by conference participants, the actionable items were presented to the CP Task Force for review and feedback. Task Force members verified the actionable items for accuracy and completeness, then collaboratively developed and finalized the research framework.

## 3. Results

### 3.1. Pre-Conference Survey (n = 36)

Thirty-six conference attendees completed the pre-conference survey. The top three priorities they identified were: communicating a CP diagnosis compassionately (mean = 4.7/5), providing realistic and helpful information to families (mean = 4.7/5), and scaling evidence-based interventions for early treatment (e.g., constraint-induced movement therapy, bimanual training, goal-directed therapy) (mean = 4.6/5). In addition, collaborators were asked to identify the most and least important research areas of concern. Thirteen of the 36 respondents rated “timing and types of early treatment” as the most important research priority. Fourteen rated “impact of early treatment on parents and parent education” as the least important.

### 3.2. Conference Focus Groups (n = 97)

The conference was held in September 2024 at a pediatric tertiary care center. Ninety-seven adults attended, including family members/caregivers, adults with CP, healthcare professionals, and additional collaborators (Table 1). Participating caregivers indicated their children represented a range of gross motor abilities across all five levels of the Gross Motor Function Classification System (I = 3, II = 3, III = 3, IV = 5, V = 1, unknown = 1). Thirty-one children were present at the conference, in childcare or as observers of keynote sessions, but they did not contribute to data collection.

Five major themes were identified from the focus group discussions. Direct quotes from the focus groups are provided in Supplementary Material S4.

Theme 1: Healthcare system barriers: Challenges identified included inconsistent referrals, limited resources, and difficulty navigating care systems. The healthcare system presents numerous barriers for individuals with CP and their families, often leading to delays in care, miscommunication, and financial strain. One major challenge is the inconsistency in referrals and disagreements between providers, which can delay access to specialists. As one clinician expressed, “It’s so unnecessary that there’s so much delay… if we could just take away that wall, and have the family have access to a specialist without the need for referral.” Families also face difficulty navigating complex systems, especially when initial concerns are dismissed, as shared by a caregiver: “They immediately said, well, we can’t diagnose until she’s like 2… but it looks like she has cerebral palsy.” Limited communication between private and school-based therapy providers further fragments care. A clinician noted, “I’m like, well what are their goals with the private OT? And they’re like we have no idea.” The lack of coordination extends to insurance issues and inconsistent diagnostic protocols, contributing to delays in necessary equipment and services. One caregiver recalled “waiting 6 months to be approved for a gait trainer or a wheelchair.” Rural families often experience even greater gaps, being “dropped off” by early intervention systems with false assurance that schools will take over, which often doesn’t meet the child’s needs. Financial burdens and long waitlists, such as for Medicaid Waiver programs, intensify the stress, with one individual stating, “I’ve been on the waiting list since 2017… I’ll be lucky if I get services… in my lifetime.” These systemic shortcomings reveal a critical need for more cohesive, accessible, and family-centered care pathways.

Theme 2: Lack of awareness and education: Participants noted gaps in public understanding and the need for informed professionals. A lack of awareness and education about CP creates meaningful challenges across public, educational, and healthcare settings, often leaving families feeling isolated and without the information they need. Public understanding remains limited, with misconceptions forming early in life. As one caregiver gently reflected, “Because when I was growing up and I was in school, all the kids that were in wheelchairs, that drooled all over themselves, also known as my daughter, I thought they had diseases, and I shouldn’t go near them because I could catch it. So, the word disease makes it seem different than diagnosis.” This gap in understanding unfortunately extends into healthcare, where some providers feel unprepared to serve patients with CP. One clinician noted, “I feel like there aren’t even enough providers, specialists across the lifespan. We hear of pediatric patients who get turned away from specialists because ‘Oh I don’t feel comfortable’ or ‘I’m not familiar with the diagnosis of CP’.” Families often find themselves navigating unfamiliar medical territory without adequate guidance, with one caregiver sharing, “I’m learning, but you know, [for] a medical doctor to ask me why I’m here, it can be so intimidating, cause you feel stupid saying ‘I have no idea; our doctor just sent us here’.” The struggle is particularly acute when families receive a diagnosis but little practical support moving forward. One parent expressed this need clearly: “Give me a solution… they start [with] ‘You have to change your life. It’s gonna be different’… But that was it.” Participants identified hopeful pathways forward, including teaching children about differences from a young age, providing resources “available in the language of the parents,” and ensuring professionals receive training that prepares them to confidently and compassionately support individuals with CP and their families throughout life.

Theme 3: Communication and compassion: Attendees emphasized the importance of empathy and clarity when discussing diagnoses and care options. The manner in which diagnoses are communicated can profoundly shape a family’s journey, with participants emphasizing the critical need for empathy, clarity, and compassionate delivery of information. Families described painful experiences when diagnoses were handled insensitively, such as one caregiver who learned about their child’s CP through an online patient portal rather than from their neurologist: “I go online and get on patient portal, of course I see the diagnosis of, you know, cerebral palsy. And I was like well, he didn’t even mention that.” The impact of negative framing was particularly striking, as illustrated by one parent’s experience: “Five people came in; some of them were interns. But the person who was actually making the presentation to us said ’She has cerebral palsy.’ And he kind of had a smirk on his face when he said it… I asked him what we could expect and his verbiage was, ‘She’ll be needing to be institutionalized. She won’t be able to feed herself…’ Well, needless to say we fought back tears.” Healthcare providers acknowledged the delicate balance required, with one clinician noting, “Don’t give too much information to the parents at once because [they] can be overwhelmed. We need to work, you know, little by little.” There was also recognition of the emotional complexity families face, as a clinician reflected, “We need to respect the process that parents, they are grieving… Those parents are dealing with a lot… Moms feel guilt. ‘What I did? Did I have the right care of myself when I was pregnant?’” Families called for a fundamental shift toward hope and normalization: “Definitely positive language and that there’s hope and disability is not a bad word; it’s not a bad thing… How do you emotionally help with that, cause we’re not just physical beings; we’re emotional beings.” This perspective reflects a desire for communication that honors both the physical and emotional dimensions of raising a child with CP, setting families up for resilience rather than despair.

Theme 4: Early intervention and therapy access: Attendees stressed the need for timely, interdisciplinary care with engaging and innovative therapeutic approaches. Access to early intervention services emerged as a critical factor in supporting children with CP, though families face significant barriers in obtaining timely, sustained, and geographically accessible care. Many caregivers spoke passionately about the transformative impact of Early Childhood Intervention (ECI), with one parent sharing, “I feel like that really helped and shaped my daughter to be in a better position than if she wouldn’t have had that. So, I honestly am a firm believer on the ECI program. That shaped my kid.” An individual with CP reflected on their own early experiences: “I like the fact that my mom had resources… where intensive therapy like Montessori classes, and PT, OT at a very early age. It was like 3 h a day, 5 days a week and repetitive, so that was hopeful in early childhood before kindergarten.” However, access remains uneven and often unsustainable. Families described traveling great distances, “We were driving an hour and a half, three days a week” and facing harsh realities when transitioning out of ECI, with one caregiver noting, “When we got out of ECI, I felt like we were thrown to the wolves… Because my insurance only covered 50 therapies a year, for my daughter. She needs more than that.” The frustration with delayed diagnosis and the “wait and see” approach was palpable, as clinicians and families alike urged, “Stop ‘the wait and see’ mentality… because we are losing precious time.” Financial constraints compound these challenges: “We’re not doing therapy because we can’t really afford it right now. We both have good jobs, but still it’s a LOT of money.” As children age, resources often diminish. One caregiver commented, “As she’s getting older, there’s also less resources… she’s going to be 14 and for two years, she hasn’t had OT.” Participants emphasized the importance of early, innovative interventions, with one clinician noting, “It’s never too early to present communication options, and it’s never too early to present the opportunity to explore one’s own environment. I’ve seen a child as young as 6 months in a power wheelchair.”

Theme 5: Infrastructure and accessibility: Advocacy for accessible community resources and policy reforms was a recurrent theme. Physical and systemic barriers to accessibility remain widespread, limiting full participation for individuals with CP across community spaces, healthcare settings, and daily life. Participants identified critical gaps in infrastructure, from inaccessible medical facilities to inadequate public spaces. One individual with CP shared a simple yet profound wish: “One thing I hope for is better access to medical services, like it would be my dream when I go to the primary doctor, that instead of asking me how much I weigh, that they would provide a scale that you could roll up.” The challenges extend to essential healthcare, with another individual recounting, “A well-known hospital turned her away because she said her wheelchair was not accessible to the equipment that they use to detect breast cancer. And like when they say ‘Hop on the table’ [laughing]… I’m just wondering ‘What medical tests are we missing?’” Public spaces also fall short, as an educator observed: “I notice that a lot of our communities… there’s a lack of equipment and things like that in public parks for people that have disabilities. I taught at a school where we didn’t have a playground that was accessible for our children in wheelchairs.” Geographic inequity compounds these issues, with caregivers emphasizing, “Just to stop having resources in one particular area. I feel like we should have resources everywhere, like the suburbs, the rural areas.” Travel presents its own set of frustrations, as one caregiver described the ordeal of air travel with a wheelchair: “They just throw the wheelchair, and they always mess up something… [One time on a trip] we got stuck in the subway at the bottom, and we had to carry her wheelchair up.” Yet participants also pointed to proof that accessibility is achievable, noting, “Morgan’s Wonderland [an accessible amusement park], everything is accessible. Everything. And it’s not that hard. When you go there, you realize wow, the world really could be accessible; they just choose not to be because it’s not cost effective.” These experiences underscore the urgent need for policy reforms and community commitment to genuine accessibility.

### 3.3. Quantitative and Qualitative Data Synthesis and Actionable Items

Based on integration of quantitative and qualitative data, with a focus on priorities that emerged as most important across data sets, actionable items were distinguished:

- Improving diagnosis communication: Emphasizing empathy and clarity when informing families about a CP diagnosis.
- Ensuring early referrals and interdisciplinary collaboration: Promoting proactive care and comprehensive networks.
- Creating inclusive education and training: Enhancing the skills and empathy of care providers while fostering self-advocacy among families.
- Scaling evidence-based therapies and researching new interventions: Expanding accessibility in proven therapeutic interventions and researching new, innovative interventions.
- Developing social support systems: Developing parent navigators, peer support groups, and advocacy networks.
- Advocating for policy and cultural change: Promoting insurance reforms, inclusive communities, and media-driven awareness campaigns.

### 3.4. Conference Follow-Up Survey (n = 16)

Sixteen conference attendees completed the follow-up survey approximately one month after the conference. This survey served primarily as a form of member checking to ensure that the identified actionable items aligned with participants’ perspectives, and secondarily to understand the conference’s impact on perspectives and approaches to care. All respondents answered ‘yes’ to the question about whether the research priorities listed were consistent with conference discussions, affirming the validity of the thematic analysis. Two respondents suggested additional priorities: nutrition therapy and transitions from pediatric to adult care. Nutrition therapy was incorporated into the broader theme of timely interdisciplinary care. While transitions from pediatric to adult care is an important topic, the primary focus of this conference was on young children, and we felt this theme was outside of the scope of the current study. Table 2 summarizes responses about whether the conference influenced participants’ perspectives, caregiving/clinical practice, and advocacy. When asked how likely they would be to recommend a similar conference (scale of 1–10), 15 collaborators chose 10 and one chose 7, indicating strong overall satisfaction with the collaborative format.

### 3.5. Development of a Framework to Address Identified Priorities

**Figure 1:**
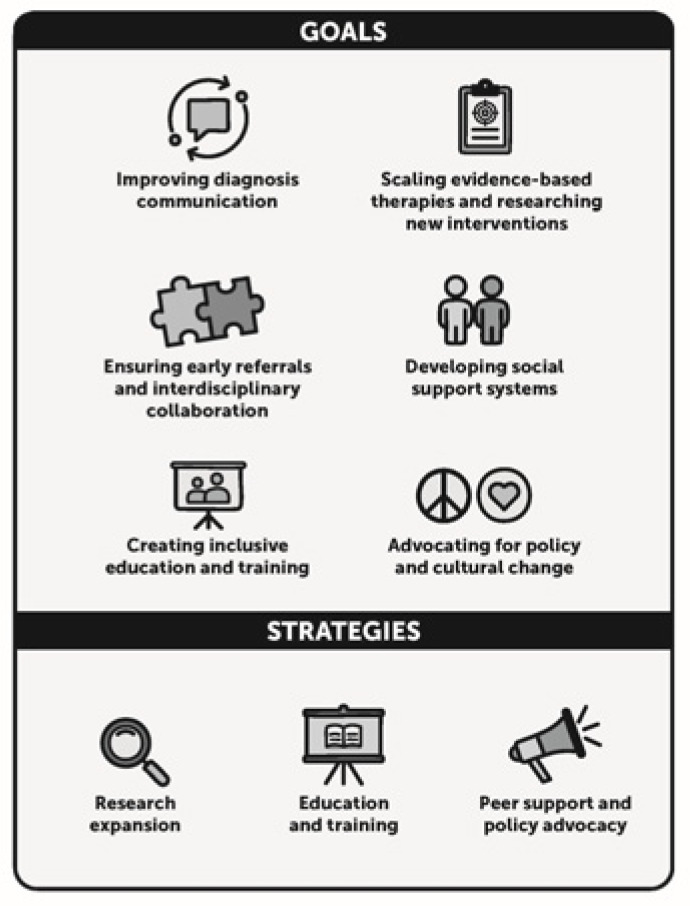
Proposed framework for advancing research and care for early cerebral palsy.

Following the conference and the conference follow-up survey, the CP Task Force collaboratively developed a research framework (Figure 1) aimed at improving care and support for children with CP, based on the priorities identified through pre-conference surveys and focus group discussions. By focusing on six actionable goals through three strategic approaches the framework ensures that identified research priorities translate into concrete action, with dedicated teams working collectively to advance one or more goals.

## 4. Discussion

The study underscores a collective aspiration to advance research and create systems that are inclusive, efficient, and family-centered for children with CP and their families. The research framework we created organizes priorities for young children with CP and their families into three strategic approaches, providing a roadmap for systemic change in CP care.

*Education and training:* The first strategy emphasizes education and training to enhance professional competencies, improve diagnosis communication with empathy and clarity, and ensure early referrals through interdisciplinary collaboration. Evidence-based guidelines already exist, including guidelines for early detection [3] and the SPIKES protocol [27] for delivering CP diagnoses to families compassionately. However, the recent literature highlights a persistent need to integrate training into academic programs and continuing education for clinicians [28,29,30,31]. Organizations such as the American Academy of Cerebral Palsy and Developmental Medicine [32], Cerebral Palsy Foundation [33], Cerebral Palsy Research Network [34], and Cerebral Palsy Alliance Research Foundation [35] actively supporting educational efforts. Utilization of low-cost multidisciplinary training models and knowledge translation strategies to promote guideline implementation has shown positive outcomes [36,37] and significant work is underway globally to implement early detection guidelines [16,38,39,40,41,42,43]. However, significant gaps remain in translating these efforts into widespread practice.

*Research expansion:* The second strategy focuses on research expansion to scale evidence-based therapies and develop innovative interventions that address current evidence gaps. Systematic reviews have established which therapeutic interventions are evidence-based for children with CP, providing clear guidance on effective practices [23]. However, significant barriers to implementation persist, including knowledge and skill gaps among practitioners [44], limited resources, and contextual factors that constrain the delivery of optimal care [45]. While existing implementation strategies such as mentoring programs, workshops, case studies, and online tools have shown promise in supporting knowledge translation [46], novel strategies are needed that will motivate both therapists and children, seamlessly integrate into busy clinical workflows, and address the real-world constraints practitioners face. Concurrently, therapeutic innovation is expanding through gamification, virtual reality, AI-based motion tracking [47], and robotics [48,49,50], though larger funding models are needed to bring these technologies to scale. Overall, there is a critical need for CP-specific funding [51,52] to accelerate gains in both therapy and implementation of best practices.

*Peer support and policy*: The third strategy centers on peer support and policy advocacy to develop social support systems and promote systemic changes that improve access to resources and empower families. Previous research has shown that families of children with CP face emotional, logistical, and financial challenges that can be alleviated through robust support systems [53,54,55,56]. For instance, family members of children with CP often report feeling isolated or overwhelmed by the demands of caregiving, and they frequently lack access to the resources they need to navigate the healthcare system. The creation of parent navigators and peer support groups addresses this gap by providing families with knowledgeable and compassionate individuals who can help guide them through the complex landscape of CP care, empowering them to advocate effectively for their children’s needs. Beyond individual support, advocacy emerged as a core theme during the focus groups, with participants emphasizing the need for systemic changes that would increase access to services, reduce financial burdens, and foster inclusive communities. This finding underscores the relevance of advocacy work in driving broader societal change. Future research should explore how to better align policies with the needs of families, how to promote inclusive community practices, and how to ensure equitable access to resources for all children with CP, regardless of socioeconomic background.

The research framework presented in this study aligns with and extends previous collaborative research priority-setting efforts in CP while offering distinct contributions to the field. Younan et al. [57] identified five priority-setting studies conducted in high-income countries with stakeholder composition similar to our sample. While the most common research priority category identified across all five studies in the Younan review was optimal intervention (61%), followed by community participation and quality of life (30%), and cause and prevention (9%), our study specifically focused on early detection and intervention, a priority area identified in three and two of those studies, respectively. One study in the Younan review explicitly noted the importance of knowledge transfer to the medical community and families about early detection/intervention, just as our participants emphasized.

The findings from this project underscore the need for an integrated, multifaceted approach to addressing the complex needs of children with CP. The framework developed can serve as a guide for future research initiatives, clinical practice improvements, and policy development. By aligning research efforts with the identified priorities, multi-collaborators can work together to create a more inclusive and effective healthcare system for children with CP. Lay summary of findings are included in Supplementary Material S5.

### Limitations

The current study reflects the perspectives of a diverse, multi-collaborator group engaged in CP care, enhancing the relevance and applicability of findings. However, generalizability is limited due to the use of convenience sampling and the recruitment of conference attendees, who may have a higher level of engagement in CP advocacy and research than the broader community. Second, the regional context of this study limits transferability, as findings from this multi-collaborator group may not fully translate to other U.S. regions or international settings where healthcare systems, resources, and cultural contexts differ. Finally, while efforts were made to foster inclusivity, certain voices may have been underrepresented.

Another limitation is the potential influence of researchers’ backgrounds and assumptions on data interpretation, despite reflexive discussions to mitigate bias. Our findings are most applicable to similar multidisciplinary, collaborator-driven settings and should be interpreted with this context in mind. Future research should include broader participant recruitment strategies and diverse data collection methods to enhance transferability.

## 5. Conclusions

This study highlights the critical need for comprehensive and compassionate care for families receiving a CP diagnosis. The priorities and framework offer a roadmap for systemic improvement in CP care across three strategies: (1) education and training to strengthen professional competencies and diagnosis communication; (2) research expansion to scale evidence-based therapies and pursue innovation; and (3) peer support and policy advocacy to build social supports and promote equitable access. Core priorities include early detection, coordinated multidisciplinary care, and well-trained providers delivering evidence-based interventions. The framework addresses the foundation needed for meaningful patient-centered research. Guided by it, collaborative efforts can ensure timely, compassionate, evidence-based care that improves outcomes and quality of life for children with CP and their families.

## Figures and Tables

**Table 1:** Conference Attendees (*n* = 97).

	*n*	%
**Gender**		
Male	22	23%
Female	75	77%
**Perspective**		
Family member/caregiver	31	32%
Adult with cerebral palsy	11	11%
Healthcare professional	39	40%
Other	16	17%
**Race ^1^**		
Native American Indian or Alaska Native	5	5%
Asian	4	4%
Black or African American	8	9%
Native Hawaiian or Other Pacific Islander	0	0%
White or Caucasian	70	72%
Two or More	5	5%
Other	3	3%
Prefer Not to Answer	2	2%
**Hispanic, Latino or Spanish Origin ^1^**		
Yes	23	24%
No	74	76%
**Preferred Language**		
English	94	97%
Spanish	3	3%

^1^ Data collected using U.S. categories specified in the Common Data Elements suggested by the National Institute of Neurological Disorders and Stroke and American Academy for Cerebral Palsy and Developmental Medicine.

**Table 2:** Response to Conference Follow-up Survey Question, “Did your participation in the conference influence change?” (*n* = 16).

Item	*n*	%
Yes, changed my perspective of cerebral palsy and early detection	12	75
Yes, changed my perspective of cerebral palsy and early intervention	9	56
Yes, changed my perspective of patient-centered outcomes research	9	56
Yes, changed my approach to caring for individuals with cerebral palsy	9	56
Yes, changed how I advocate for individuals with cerebral palsy	11	69
No	2	13
Other	1	1

## Data Availability

The data supporting this study’s findings are not publicly available. Data may be made available upon reasonable request to the corresponding author, subject to Institutional Review Board approval and adherence to confidentiality agreements.

## Supplementary Materials

The following supporting information can be downloaded at: https://www.mdpi.com/article/10.3390/jcm14217592/s1, File S1: Pre-conference Research Priority Rating Survey; File S2: Focus Group Moderator Guide; File S3: Conference Follow-Up Survey; File S4: Direct Quotes from Focus Groups; File S5: Lay Summary.

## Abbreviations

The following abbreviations are used in this manuscript:

CIMT	constraint-induced movement therapy
CP	cerebral palsy
DFW	Dallas–Fort Worth
ECI	early childhood intervention
G-button	gastrostomy button
HCS	Home and Community-based Services
IDD	intellectual and developmental disabilities
OT	occupational therapy
PT	physical therapy/physical therapist
