# Supplementary material for: Multi-Collaborator Engagement to Identify Research Priorities for Early Intervention in Cerebral Palsy
Source: J Clin Med. 2025 Oct 26;14(21):7592. doi: 10.3390/jcm14217592 (PMC12610828; doi:10.3390/jcm14217592)
Supplement: Supplementary file 1 [file jcm-14-07592-s001.zip › Supplementary Material S1.pdf]

Pre-Conference Research Priority Rating Survey

Please complete the survey below.

Thank you!

This survey is about rating how important different research ideas are for studying cerebral palsy in young children. Rate each topic from 1 to 5, with 1 being not important and 5 being very important.

[Attachment: "STAMPED STU2024-0022, Shierk, FormE.I-InfoSheet, Mod_1, 07-29-2.pdf"]

____

Early Detection and Diagnosis

---

1) Use guidelines that help doctors detect and diagnose cerebral palsy in babies 3 to 6 months old, including using standard tests.

- ◯ 1: Not at all important
- ◯ 2: Slightly important
- ◯ 3: Moderately important
- ◯ 4: Important
- ◯ 5: Very important

---

2) We need to make clear rules for what "at risk" means when talking about cerebral palsy.

- ◯ 1: Not at all important
- ◯ 2: Slightly important
- ◯ 3: Moderately important
- ◯ 4: Important
- ◯ 5: Very important

---

3) Find ways to tell parents their child has cerebral palsy in a caring way that helps.

- ◯ 1: Not at all important
- ◯ 2: Slightly important
- ◯ 3: Moderately important
- ◯ 4: Important
- ◯ 5: Very important

---

4) Identify ways doctors and therapists can give parents realistic information about what to expect as they raise a child with CP.

- ◯ 1: Not at all important
- ◯ 2: Slightly important
- ◯ 3: Moderately important
- ◯ 4: Important
- ◯ 5: Very important

---

5) Additional ideas for research on early detection and diagnosis of cerebral palsy:

____

Working Together to Start Early Treatment

---

6) There is a difference between what help parents of young children with CP want compared to what help they actually get. We need to study this.

- ◯ 1: Not at all important
- ◯ 2: Slightly important
- ◯ 3: Moderately important
- ◯ 4: Important
- ◯ 5: Very important

7) Create a "100 day kit" for families when their child is first diagnosed. This kit would have information they need to take care of their child in the first 100 days after receiving the diagnosis. It would include information about movement, feeding, participating in activities, and how parents can take care of themselves.

◯ 1: Not at all important
◯ 2: Slightly important
◯ 3: Moderately important
◯ 4: Important
◯ 5: Very important

8) Do more studies on personal care plans, coordinating care, and the role of the main pediatric providers for young children with CP.

◯ 1: Not at all important
◯ 2: Slightly important
◯ 3: Moderately important
◯ 4: Important
◯ 5: Very important

9) Create guidelines for doctors and therapists caring for babies under 2 years old with CP.

◯ 1: Not at all important
◯ 2: Slightly important
◯ 3: Moderately important
◯ 4: Important
◯ 5: Very important

10) Additional ideas for research on working together to start early treatment:

____

Early Treatment - Timing and Types of Help

11) Learn when early treatments should start for young children with CP.

◯ 1: Not at all important
◯ 2: Slightly important
◯ 3: Moderately important
◯ 4: Important
◯ 5: Very important

12) Study how to use the brain's ability to change to improve movement, speech, and mobility.

◯ 1: Not at all important
◯ 2: Slightly important
◯ 3: Moderately important
◯ 4: Important
◯ 5: Very important

13) Learn the long term results of treatments started before age 2.

◯ 1: Not at all important
◯ 2: Slightly important
◯ 3: Moderately important
◯ 4: Important
◯ 5: Very important

14) Find ways like scans, blood tests, and spinal fluid tests, to help doctors know which treatments will work best for a young child with CP.

◯ 1: Not at all important
◯ 2: Slightly important
◯ 3: Moderately important
◯ 4: Important
◯ 5: Very important

15) Study how treatment affects thinking and learning abilities in young children with CP.

◯ 1: Not at all important
◯ 2: Slightly important
◯ 3: Moderately important
◯ 4: Important
◯ 5: Very important

16) Study ways to use proven treatments like constraint movement therapy, training both hands together, and goal-directed training on a larger scale in early treatment.

- ◯ 1: Not at all important
- ◯ 2: Slightly important
- ◯ 3: Moderately important
- ◯ 4: Important
- ◯ 5: Very important

Early Treatment - Impact on Parents and Parent Education

17) Learn how the type and amount of therapy affects parents.

- ◯ 1: Not at all important
- ◯ 2: Slightly important
- ◯ 3: Moderately important
- ◯ 4: Important
- ◯ 5: Very important

18) Find the best ways to teach parents how to use toys and other items in their home to help their child learn and become more independent.

- ◯ 1: Not at all important
- ◯ 2: Slightly important
- ◯ 3: Moderately important
- ◯ 4: Important
- ◯ 5: Very important

19) Find the best ways to provide proven information on the importance of good sleep and eating.

- ◯ 1: Not at all important
- ◯ 2: Slightly important
- ◯ 3: Moderately important
- ◯ 4: Important
- ◯ 5: Very important

20) Determine if family coaching improves care.

- ◯ 1: Not at all important
- ◯ 2: Slightly important
- ◯ 3: Moderately important
- ◯ 4: Important
- ◯ 5: Very important

21) Find the best ways to provide proven information to encourage parent involvement in therapy including formal tools to set goals and giving parents illustrated home programs.

- ◯ 1: Not at all important
- ◯ 2: Slightly important
- ◯ 3: Moderately important
- ◯ 4: Important
- ◯ 5: Very important

22) Additional research ideas related to early treatment:

____

23) Select the area that is most important to you

- ◯ Research on early detection and diagnosis of Cerebral Palsy
- ◯ Research on working together to start treatment early
- ◯ Research on the timing and types of early treatment
- ◯ Research on the impact of early treatment on parents and parent education

24) Select the area that is least important to you

- ◯ Research on early detection and diagnosis of Cerebral Palsy
- ◯ Research on working together to start treatment early
- ◯ Research on the timing and types of early treatment
- ◯ Research on the impact of early treatment on parents and parent education
