# Supplementary material for: Multi-Collaborator Engagement to Identify Research Priorities for Early Intervention in Cerebral Palsy
Source: J Clin Med. 2025 Oct 26;14(21):7592. doi: 10.3390/jcm14217592 (PMC12610828; doi:10.3390/jcm14217592)
Supplement: Supplementary file 1 [file jcm-14-07592-s001.zip › Supplementary Material S2.pdf]

Focus Group Moderator Guide - Session 1 Early Detection and Diagnosis and Initiation of Early Intervention (75 minute session)

Preparation

Room Setup (laptop to record)

Jamboard:
https://jamboard.google.com/d/1XG8i0iyupMDrXq7mR7mwbVQbqXi6XhRaFu9geXbfjg4/edit?usp=sharing

- Arrange seating in a circle or around a table to encourage discussion
- Ensure all participants can see and hear each other

Welcome and Introduction (5 minutes)

- Greet participants as they arrive

Opening Remarks (5 minutes)

- Introduce yourself as the moderator and any co-facilitators or assistants
- Explain the purpose of the focus group.
- Review the agenda and ground rules

Example Script: "Welcome, everyone. My name is (Your name), and I will be facilitating today's focus group. The purpose of our discussion is to gather information about your experiences with Early Detection and Diagnosis and the Initiation of Early Intervention. Your insights will help us set research priorities for young children with CP. We have a series of questions to guide our discussion and we encourage open and honest comments. There are no right or wrong answers.

- Ground Rules

Example Script: "The CP task force has set team norms you see on the table. We ask that you remember 1. All team members are equal. 2. All team members are valued and encouraged to fully participate. 3. All team members agree that the team's established norms will guide their behavior on the team. 4. Team members agree to assess their own behavior as it pertains to the established norms. 5. Speak openly about ideas, perspectives and experiences without judgement. 6. Speak respectfully to each other (not talk down, not interrupt, recognize and thank).

Participants

- You were selected because you have CP, or are a family member with CP, a physician or healthcare provider that works with children with CP, an administrator, student, faculty member that has a stake in the care of children with CP.
- Guidelines
    - No right or wrong answers, only differing points of view
    - We're recording, one person speaking at a time
    - We invite you to turn your cameras on for the discussion, but it is not required to participate.(online)
    - We're on a first name basis

- You don't need to agree with others, but you must listen respectfully as others share their views
- We ask that you turn off your phones or other electronic devices to minimize distractions. If you cannot and if you must respond, please do so as quietly as possible and rejoin us as quickly as you can.
- My role as moderator will be to guide the discussion

Discussion Guide (5 minutes)

1. **Icebreaker/ Opening Question**

- *Moderator Instructions*: The questions are easy to answer, nonthreatening, and surround your experiences as an individual with CP or as a stakeholder. The icebreaker also creates a climate where the **moderator goes in a circle** to make sure that each participant speaks out loud in front of the rest of the group; this action makes it easier for people to speak again during the remainder of the session. Going around the circle allows the moderator to establish a welcoming and inclusive feel to the group discussion by validating that everyone's voice is important to the discussion, regardless of how benign the icebreaker questions might seem.

- *Moderator Script*: (30 seconds per person)
  - Briefly tell us a little about yourself including where you are from, the perspective you bring to the conference, and why you wanted to be part of this conference.

2. **Introductory Question (10 minutes)**

- *Moderator Instructions:* This is where a topic is introduced that is related to the session topic but is still general enough to be easy to answer, nonthreatening, and encourages participants to contribute to **another round of speaking out loud** and getting used to speaking in the group. This topic may cover a connection with the topic in some way, such as "tell us, in a minute to a minute and a half, about a phrase or expression you would use to describe how you feel about teaching college students."

- *Moderator Script:* (60 seconds per person)
  - Tell us, in about a minute, what comes to mind when you think on a detective or a sleuth? Follow up prompts could be - your favorite detective, favorite TV show or movie that involves detectives or solving a mystery, have you ever had to be a 'detective'? What qualities make up a good detective or sleuth?

3. **Transition Instructions (5 minutes)**

- *Moderator Instructions:* This is where the discussion moves more specifically to the topic under study. In this phase, the general, broad conversation topics become more focused and more personal for participants. This is also the first time in the discussion when the moderator **lets participants speak on their own impulses**, rather than creating a structure for everyone to speak; it is the beginning of moving

from a moderator-directed conversation to a participant-directed conversation, so the question must generate that inclination for participants. Questions are directly related to the session topic, and the **moderator uses probes** to encourage rich, detailed examples and descriptions of the participants' experiences.

- *Moderator Script:* (about 1 minute per person responses)
  - For each of the breakout sessions we will be creating chain links made up of red, yellow, green, and purple links.
    - The red links represent practices or situations that do not result in positive outcomes that you would like to see stopped. Red = Stop
    - The yellow links will represent practices or situations that may or may not result in positive outcomes and should be approached with caution. Yellow = Yield or proceed with caution
    - The green links represent practices or situations that result in positive outcomes and should be used. Green = Go
    - The purple links represent what you hope for related to out topic of Early Detection and Diagnosis for cerebral palsy, Purple = Hope
  - Prior to the conference, you completed a survey ranking the importance of research priorities related to this topic.
  - These are the results of the survey (share results on screen)
  - Based on your experiences, fill out one of the chain links related to early detection and diagnosis of cerebral palsy. What you write down can be related to the research priorities that we just reviewed or you can write down a new idea.
  - You will have the opportunity to share what you wrote on your chain links as we discuss early detection and diagnosis of cerebral palsy.
    - The goal of this activity is to get them thinking about research priorities related to early detection and diagnosis and their own lived experiences around this topic and prepare to discuss them.
  - Thank you for writing down some of your thoughts. As we continue our discussion, continue to write ideas on the chain links. The moderator of the session will also be writing down ideas on the chain links based on our conversation.

4. **Key or Content Questions (40 minutes)**

- *Moderator Instructions:* This is where the real work of the focus group happens. The questions posed in this part of the discussion anchor the entire discussion; **at least three or four substantial questions are asked by the moderator**, and sometimes there might even be four to six questions. However, it is unlikely that more than four or five questions can be asked of participants without sacrificing the necessary details and stories that should come with participant perspectives. Asking too many questions may mean that insufficient details are provided, since participants may feel rushed by the moderator or may feel that there is not ample time for everyone to

contribute to the discussion. Careful crafting of content questions is essential to answering your research questions and building a sense of synergy and ease in your group.

- *Moderator Script:* (open-ended, entire segment comprises approximately 40 minutes of the 75-minute focus group)
  - Can you describe positive experiences or processes that are in place related to early detection and diagnosis (early or late) – green
  - Can you describe positive experiences or processes that are in place related to the initiation of early intervention (early or late) - green

  - Can you describe experiences or processes that were ok but not ideal (yellow) or that you would recommend being discontinued (red) related to early detection or diagnosis (early or late)?
  - Can you describe experiences or processes that were ok but not ideal (yellow) or that you would recommend being discontinued (red) related to the initiation of intervention (early or late)?
  - Describe what you hope for or possible solutions for the research priorities we discussed during the session including early detection and diagnosis of CP and the initiation of early intervention (purple).

5. **Debriefing/ Concluding Question (5 minutes)**

- Moderator Instructions: This is where the moderator determines that after the key questions have been covered, the **group must exit the discussion safely and comfortably**. There may be times when participants are deeply affected by a discussion or when their emotions or memories are disturbed in some way; creating a safe space within which participants can debrief, unload feelings, or process the discussion is another important element in focus group research. The questions created for this final phase should acknowledge the discussion that has just occurred but also return to a general level of discussion to depart from the intensity or intimacy of the main discussion.

- Moderator Script:
- Thank participants for their time and contributions
  Example Script: "Thank you all for your time and valuable insights. Your feedback is crucial in helping us develop research priorities for young children with CP.
  - We anticipate that great conversation will occur along with the identification of gaps/problems with solution-focused recommendations. This would end the discussion with positive, hopeful next steps.
  - Help attendees move to the next part of the conference (group picture)

Collect all notes and ensure secured safely with the recordings
Meet with co-facilitators to discuss initial impressions and any immediate observations.

Tips for Facilitators

1. Active listening
   a. Show interest through nodding and maintaining eye contact
   b. Paraphrase or summarize points to ensure understanding
2. Encourage Participation
   a. Invite quieter participants to share their thoughts
3. Manage Dynamics
   a. Address dominant participants by gently steering the conversation
   b. Ensure respectful and constructive feedback
4. Stay Neutral
   a. Avoid sharing your opinions or leading participants toward a specific viewpoint
5. Keep on Track
   a. Be mindful of time and ensure all questions are covered.

References

Billus, F. D. (2021). *Qualitative data collection tools: Design, development, and applications.* (Qualitative Research Methods Book 55), Sage Publications.

Krueger, R. (2002, October). *Designing and conducting focus group interviews.* https://www.eiu.edu/ihec/Krueger-FocusGroupInterviews.pdf

Focus Group Moderator Guide - Session 2 Early Treatment and Community Resources (Timing, Types, and Parents)

Preparation

Room Setup (online set zoom link)

- Arrange seating in a circle or around a table to encourage discussion
- Ensure all participants can see and hear each other

Welcome and Introduction (5 minutes)

- Greet participants as they arrive

Opening Remarks (5 minutes)

- Introduce yourself as the moderator and any co-facilitators or assistants
- Explain the purpose of the focus group.
- Review the agenda and ground rules

Example Script: "Welcome, everyone. My name is (Your name), and I will be facilitating today's focus group. The purpose of our discussion is to gather information about your experiences with Early Treatment. Your insights will help us set research priorities for young children with CP. We have a series of questions to guide our discussion and we encourage open and honest comments. There are no right or wrong answers.

- As we have already met this morning for the first and second breakout session, I will give an overview of the ground rules

Example Script: "The CP task force has set team norms you see on the table. We ask that you remember 1. All team members are equal. 2. All team members are valued and encouraged to fully participate. 3. All team members agree that the team's established norms will guide their behavior on the team. 4. Team members agree to assess their own behavior as it pertains to the established norms. 5. Speak openly about ideas, perspectives and experiences without judgement. 6. Speak respectfully to each other (not talk down, not interrupt, recognize and thank).

Participants

- You were selected because you have CP, or are a family member with CP, a physician or healthcare provider that works with children with CP, an administrator, student, faculty member that has a stake in the care of children with CP.
- Guidelines
    - No right or wrong answers, only differing points of view
    - We're recording, one person speaking at a time
    - We invite you to turn your cameras on for the discussion, but it is not required to participate.(online)
    - We're on a first name basis

- You don't need to agree with others, but you must listen respectfully as others share their views
- We ask that you turn off your phones or other electronic devices to minimize distractions. If you cannot and if you must respond, please do so as quietly as possible and rejoin us as quickly as you can.
- My role as moderator will be to guide the discussion

Discussion Guide

1. **Transition Instructions (5 minutes)**

- *Moderator Instructions:* This is where the discussion moves more specifically to the topic under study. In this phase, the general, broad conversation topics become more focused and more personal for participants. This is also the first time in the discussion when the moderator **lets participants speak on their own impulses**, rather than creating a structure for everyone to speak; it is the beginning of moving from a moderator-directed conversation to a participant-directed conversation, so the question must generate that inclination for participants. Questions are directly related to the session topic, and the **moderator uses probes** to encourage rich, detailed examples and descriptions of the participants' experiences.

- *Moderator Script:*
  - We will create the chain links during our discussion like we did during the first and second session. As a reminder
    - The red links represent practices or situations that do not result in positive outcomes that you would like to see stopped. Red = Stop
    - The yellow links will represent practices or situations that may or may not result in positive outcomes and should be approached with caution. Yellow = Yield or proceed with caution
    - The green links represent practices or situations that result in positive outcomes and should be used. Green = Go
    - The purple links represent what you hope for related to our topic of Early Treatment for cerebral palsy, Purple = Hope
  - Prior to the conference, you completed a survey ranking the importance of research priorities related to this topic.
  - These are the results of the survey (share results on screen)
  - Based on your experiences, fill out one of the chain links related to early treatment of cerebral palsy. What you write down can be related to the research priorities that we just reviewed or you can write down a new idea.

- You will have the opportunity to share what you wrote on your chain links as we discuss early treatment.

    - The goal of this activity is to get them thinking about research priorities related to early detection and diagnosis and their own lived experiences around this topic and begin to discuss them.
- Thank you for writing down some of your ideas - as we continue our discussion, continue to write ideas on the chain links. The moderator of the session will also be writing down ideas on the chain links based on our conversation.

2. **Key or Content Questions**

- *Moderator Instructions:* This is where the real work of the focus group happens. The questions posed in this part of the discussion anchor the entire discussion; **at least three or four substantial questions are asked by the moderator**, and sometimes there might even be four to six questions. However, it is unlikely that more than four or five questions can be asked of participants without sacrificing the necessary details and stories that should come with participant perspectives. Asking too many questions may mean that insufficient details are provided, since participants may feel rushed by the moderator or may feel that there is not ample time for everyone to contribute to the discussion. Careful crafting of content questions is essential to answering your research questions and building a sense of synergy and ease in your group.

- *Moderator Script:* (open-ended, entire segment comprises approximately 20 minutes of the 60-minute focus group)
    - Can you describe positive experiences or processes that are in place related to early treatment (early or late) - green
    - Can you describe experiences or processes that were ok but not ideal (yellow) or that you would recommend be discontinued (red) related to early treatment?
    - Describe what you hope for or possible solutions for the research priorities we discussed today.

3. **Debriefing/ Concluding Question (10 minutes)**

- Moderator Instructions: This is where the moderator determines that after the key questions have been covered, the **group must exit the discussion safely and comfortably**. There may be times when participants are deeply affected by a discussion or when their emotions or memories are disturbed in some way; creating a safe space within which participants can debrief, unload feelings, or

process the discussion is another important element in focus group research. The questions created for this final phase should acknowledge the discussion that has just occurred but also return to a general level of discussion to depart from the intensity or intimacy of the main discussion.

- Moderator Script:
- Thank participants for their time and contributions
  Example Script: "Thank you all for your time and valuable insights. Your feedback is crucial in helping us develop research priorities for young children with CP.
    - We anticipate that great conversation will occur along with the identification of gaps/problems with solution focused recommendations. This would end the discussion with a positive, hopeful next steps.
    - Help attendees move to the next part of the conference: resource fair or back to Reverchon for dance performance

Collect all notes and ensure secured safely with the recordings
Meet with co-facilitators to discuss initial impressions and any immediate observations.

Tips for Facilitators

1. Active listening
   a. Show interest through nodding and maintaining eye contact
   b. Paraphrase or summarize points to ensure understanding
2. Encourage Participation
   a. Invite quieter participants to share their thoughts
3. Manage Dynamics
   a. Address dominant participants by gently steering the conversation
   b. Ensure respectful and constructive feedback
4. Stay Neutral
   a. Avoid sharing your opinions or leading participants toward a specific viewpoint
5. Keep on Track
   a. Be mindful of time and ensure all questions are covered.

References

Billus, F. D. (2021). *Qualitative data collection tools: Design, development, and applications.* (Qualitative Research Methods Book 55), Sage Publications.

Krueger, R. (2002, October). *Designing and conducting focus group interviews.* https://www.eiu.edu/ihec/Krueger-FocusGroupInterviews.pdf

SCOTTISH RITE FOR CHILDREN®

THE CASE OF THE MISSING RESEARCH PRIORITIES FOR YOUNG CHILDREN WITH CEREBRAL PALSY

Summary of Research Priorities

Early Detection and Diagnosis of Cerebral Palsy and Starting Intervention Early
Top Three Based on The Pre-Conference Survey

1. Use guidelines that help doctors detect and diagnose cerebral palsy in babies 3 to 6 months old, including using standard tests.

2. Find ways to tell parents their child has cerebral palsy in a caring way that helps.

3. Create guidelines for doctors and therapists caring for babies under 2 years old with CP.

Summary of Research Priorities

Early Intervention
Top Three Based on Pre-Conference Survey Results

1. Study how to use the brain's ability to change to improve movement, speech, and mobility.
2. Study ways to use proven treatment like constraint-induced movement therapy, training both hands together, and goal-directed training on a larger scale in early treatment.
3. Find the best ways to teach parents how to use toys and other items in their home to help their child learn and become more independent.

Rated as most important: research on timing and types of intervention

Rated as least important: research on the impact of early treatment on parents and parent education

Thank You

SCOTTISH RITE
FOR CHILDREN ®
