# Supplementary material for: Multi-Collaborator Engagement to Identify Research Priorities for Early Intervention in Cerebral Palsy
Source: J Clin Med. 2025 Oct 26;14(21):7592. doi: 10.3390/jcm14217592 (PMC12610828; doi:10.3390/jcm14217592)
Supplement: Supplementary file 1 [file jcm-14-07592-s001.zip › Supplementary Material S3.pdf]

Conference Follow Up Survey

Please complete the survey below.

Thank you!

The following research priorities were identified during the conference on cerebral palsy and early intervention.

1. Early referrals and proactive care from birth including
a) building a network of interdisciplinary providers
b) fun and engaging therapies
c) use of technology

2. Social support including
a) parent advocates/navigators
b) peer support groups

3. Need for advocacy including
a) access to resources
b) insurance that empowers families
c) change to environment vs change to person
d) greater awareness through media
e) inclusive communities and mindset

4. Education and Training that supports
a) well-informed and compassionate care providers
b) greater self-advocacy for families
c) training therapists and physicians on evidence-based therapy interventions specific to CP

5. Overall more research to support evidence-based interventions
a) implementation of known interventions - constraint-induced movement therapy, bimanual therapy, goal-directed therapy
b) new innovations

---

1) Do you feel these research priorities are consistent with the discussions and views shared during the conference?

○ yes
○ no

---

2) Add comments about the research priorities

____

---

3) Are there any research priorities that you would like to change or add?

○ yes
○ no

---

4) Add comments about changing of adding research priorities

____

5) Did your participation in the conference influence change? (select all that apply)

- ☐ Yes, changed my perspective of cerebral palsy and early detection
- ☐ Yes, changed my perspective of cerebral palsy and early intervention
- ☐ Yes, changed my perspective of patient centered outcomes research
- ☐ Yes, changed my approach to caring for individuals with cerebral palsy
- ☐ Yes, changed how I advocate for individuals with cerebral palsy
- ☐ No
- ☐ Other

6) Add comments about the conference's influence on change

____

7) If this conference were held again, on a scale of 1 (low) to 10 (high), how likely are you to recommend it to a friend or colleague?

- ◯ 1
- ◯ 2
- ◯ 3
- ◯ 4
- ◯ 5
- ◯ 6
- ◯ 7
- ◯ 8
- ◯ 9
- ◯ 10
