# Supplementary material for: Multi-Collaborator Engagement to Identify Research Priorities for Early Intervention in Cerebral Palsy
Source: J Clin Med. 2025 Oct 26;14(21):7592. doi: 10.3390/jcm14217592 (PMC12610828; doi:10.3390/jcm14217592)
Supplement: Supplementary file 1 [file jcm-14-07592-s001.zip › Supplementary Material S4.pdf]

Supplementary Material S4**: Direct Quotes from Focus Groups**

**Healthcare System Barriers Identified During Focus Groups**

| |
|---|
| Group 1: "For me personally, I know that when my daughter. I noticed her not having the same like movements, as my other three boys. I have three other children. And so when I went to the doctor, they immediately said, well, we can't diagnose until she's like 2 I think, but it looks like she has cerebral palsy." (caregiver) |
| Group 1: "So being able to access all that before even speaking to someone, how to do this, was difficult. So maybe thinking about before they disclose everything to parents online, discussing things with them." (caregiver) |
| Group 1: "It's so unnecessary that there's so much delay in being able to see your specialist because I mean it's up to a pediatrician and sometimes they disagree with the specialist who already told them 'we need to have this child be seen by a neurologist' but the pediatrician doesn't agree. I feel like if we could just take away that wall, and have the family have access to a specialist without the need for referral." (clinician) |
| Group 2: "So I have kids that I also do therapy for, and I'm like, well what are their goals with the private OT? And they're like we have no idea. I'm like, well what do they work on when they go three times a week? I have no idea; I sit in the parking lot because we're not allowed to go in. So they don't know how to tell us what they're working on for us to incorporate in our sessions as well or to kind of piggyback off of it." (clinician) |
| Group 2: "Insurance companies don't understand my super specific coding. So I think right there is an opportunity to make sure we're all speaking the same language because there's also cases I see here at the surgical hospital where they have the diagnosis of cerebral palsy but if I find them a more specific diagnosis, we know to give them blood, we know to do other things. And so creating that joint protocol, that to me is probably what's really important." (clinician) |
| Group 4: "So ECI is like 'Oh good; we have help. We have other people that are gonna come help us. Unfortunately, not always for you, but for other rural areas. And then you're just dropped off, and they're like 'Oh school's gonna take care of it'. And school does not take care of nothing." "I was gonna say, she gets the therapy at school but it's not what she really needs." Moderator: "So like that transition?" "Mhm." (caregiver) |
| Group 4: "We thought when our kid was going to therapy that we could put it all on credit cards. But we're still trying to pay off the last few years that we did, so we've been all over for a few years just trying to take a break to catch up so we can get started back." (caregiver) |
| Group 4: "The waiting for things to be done too. Not just the waitlist, but waiting 6 months to be approved for a gait trainer or a wheelchair." (caregiver) |
| Group 4: "And I think the waiting on the doctors too. It seems like to me, like 8 years ago getting into a neurologist took maybe like 8 weeks, and now it's like 'oh, we'll see you in May because that's the first appointment we have available. We could put you on a waitlist and see if anything becomes available." (clinician) |
| Group 7: "I have invited many family to do the things that my son is doing and put in there because they don't know the resource is available because the language sometimes they cannot go to Internet and read in English." (caregiver) |
| Group 8: "I have also been in the situation of Information Referral, and the saddest phone call I get is 'My child is 50; what's gonna happen when I'm no longer here?' So I put it on the ECI professionals to make sure families are on the waiting lists for the Medicaid Waiver programs. It |

| |
|---|
| is okay if 10, 12 years later they don't need the programs, but it is absolutely doing a disservice to not make sure they make that phone call and put the person on the list." (caregiver) |
| Group 8: "There's a problem, though, because my dear friend here, she got on there, and she recommended that I put myself on there. Well I've been on the waiting list since 2017 or something and there is like a thousand people ahead of me, and I'll be lucky if I get services for those programs in my lifetime." (individual with CP) |

**Abbreviation:** ECI, early childhood intervention.

**Comments About Lack of Awareness and Education During Focus Groups**

| |
|---|
| Group 1: "So I think doctors need to stop just pushing us through the system and giving us more information when it comes to like our own personal care. So that's one thing that I do wish to stop and that's way before even this happens to a child." (individual with CP) |
| Group 1: "Well, she was aspirating on her formula. I didn't know what aspiration was at that point, so we had to immediately, we had to go and get an emergency G-button. So I'm thankful for ECI for that, but I wouldn't have known to look for that. How was I supposed to know that my about to be CP daughter couldn't swallow properly." "Like, look for aspiration. Look for you know if she's hitting these milestones, if sleeping's an issue. I would have devoured all that information if given to me ahead of time." (caregiver) |
| Group 1: "I'm learning, but you know, like go for a medical doctor to ask me why I'm here, it can be so intimidating, cause you feel stupid saying 'I have no idea; our doctor just sent us here'." (caregiver) |
| Group 1: "Because when I was growing up and I was in school, all the kids that were in wheelchairs, that drooled all over themselves, aka my daughter, I thought they had diseases, and I shouldn't go near them because I could catch it. So the word disease makes it seem different than diagnosis." (caregiver) |
| Group 2: "What we found with my daughter is with her specific diagnosis, nobody has a clue what that is. So we weren't getting services that we needed. And as soon as we put CP on there, they were like oh, now we know what to do. So it was very eye-opening." (caregiver) |
| Group 4: "But you know, I do think there are kids that are being underprivileged; they're not being served right because…" Interjection: "Because their momma ain't hollering like you are." Interjection 2: "Parents don't know…" Interjection: "Yeah, they don't know what resources they could have." (caregivers) |
| Group 4: "It's like they haven't evolved at all. We have so many special needs and disability people in the world, and they haven't evolved. I know Delta's working on where you can bring your wheelchair and park it and walk it in, but really two seats on the whole plane? Two seats?" "And how they want you to carry your child down the plane." (caregiver) |
| Group 4: "Acceptance everywhere." "Teach your kids at a young age about other kids with disabilities because the staring – it just drives me crazy. And it's not their fault; they don't know, right? They don't know, but if parents would teach them kindness, and tell them like 'Look'. Expose them more so that they know how to handle situations better as they grow up." (caregiver) |
| Group 6: "Give me a solution. You cannot say this is what you have to do, like they start on that 'You have to change your life. It's gonna be different. You're gonna have to have different care'. But that was it. There was no... Hey, give me something that I can live with. I don't have anybody. I don't know anybody with cerebral palsy, I don't know anything about it. So in five, ECI came. I think why are they here? I don't know what this means. They were over here to |

| |
|---|
| help. Yeah, but I don't know what it means. I didn't know. Like if I have known what I know now, it'd have been like full control, full therapy when she was really young." (caregiver) |
| Group 7: "As somebody mentioned, put resources available in the language of the parents." (caregiver) |
| Group 8: "She always told me that having that parent perspective, what to expect next. And it seems like for me, I was looking for, my parents were always looking for somebody that had been through it. So they could go and ask, 'What will it be like at age 16 or 18 or whatever.' And they could not find it. So that's basically why I try to help the younger generation, the families now, because I didn't have that when I was growing up." (individual with CP) |
| Group 8: "I feel like there aren't even enough providers, specialists across the lifespan. We hear of pediatric in and out patients who get turned away from specialists because 'Oh I don't feel comfortable' or 'I'm not familiar with the diagnosis of CP' or 'I don't have experience with this' or 'Your insurance won't pay for the specialist referral' or 'The appointment's five months away'." (clinician) |

**Abbreviations:** CP, cerebral palsy; ECI, early childhood intervention; G-button, gastrostomy button.

**Comments About Communication and Compassion During Focus Groups**

| |
|---|
| Group 1: "So it was a long wait for an MRI and the neurologist just didn't tell me any diagnosis, anything, but when I go online and get on patient portal, of course I see the diagnosis of, you know, cerebral palsy. And I was like well, he didn't even mention that." "I'm like you know you're gonna give a diagnosis, you really need to let the parents know that you're going to give that because they can access that online now." (caregiver) |
| Group 1: "The typical questionnaires, I get it that they need to do those things. So that's why that's in the yellow and not the red. I get that they need to do that to find out what level they're at, but when you see a child that's just laying there, not being able to move at all like don't tell me if she can run, like could she run? She's like a turtle on her back. Do you think she can run? No. So those things are a little frustrating for me. Proceed with caution because Imma bite your head off." (caregiver) |
| Group 1: "Our pediatrician wasn't being honest with us. We were totally lost, so everywhere we went we were just getting vague..." (caregiver) |
| Group 2: "I probably out of training wasn't using the words 'cerebral palsy' enough, and she presented that right, like the last speaker, it said hypoxic ischemic injury, and in my mind I'm like well that's a diagnosis. You're gonna need services, you're gonna need help. So what I said is we really need to bridge the language gap between our wonderful families, patients, therapists. Because I didn't realize that therapists had tailored therapy for the words 'cerebral palsy'. I didn't realize when they presented that if you say 'holoprosencephaly', this doesn't have a tailored treatment for that." (clinician) |
| Group 7: "One of the things that I will recommend to have caution regarding to early diagnosis and treatment is don't give too much information to the parents at once because [they] can be overwhelmed. We need to work, you know, little by little, to see how much that parent is understanding." (clinician/researcher) |
| Group 7: "So we need to respect the process of that parents. They are grieving. I mean I am psychologist so I always go to the mental health area, but we need to be aware that those parents are dealing with a lot. Today, when the presenter was talking, I was thinking about the parents a lot. They want to know what caused this and it's because they feel guilt. Moms feel guilt. 'What I did? Did I have the right care of myself when I was pregnant? What did I did wrong? Did I pass this to my child?'" (clinician/caregiver) |

| |
|---|
| Group 8: "Maybe the doctors just didn't want you to have high expectations or something but it was negative. It was a negative experience." (caregiver) |
| Group 8: "Our daughter was 15 months old. We knew there might be something wrong with her because when she crawled, she never got up on her haunches. So we took her for an evaluation. Five people came in; some of them were interns. But the person who was actually making the presentation to us said she has cerebral palsy. And he kind of had a smirk on his face when he said it.… And I said, I grew up with a fella next door who had cerebral palsy but he couldn't do anything, and he was a case, if you will. So that was my version of cerebral palsy; I thought of that man. I asked him what we could expect and his verbiage was 'She'll be needing to be institutionalized. She won't be able to feed herself and she won't be able to do this or that. Well, needless to say we fought back tears." (caregiver) |
| Group 8: "Definitely positive language and that there's hope and disability is not a bad word; it's not a bad thing. I mean, I would like there to be language that's used more that normalizes disability because if you live long enough, you're gonna have a disability. It's normal. So early on, have that language for the parents because how are the parents supposed to help the child adapt to being a normal child but then having leg braces or crutches. How do you emotionally help with that, cause we're not just physical beings; we're emotional beings. And to be able to access not only the physical environment, but also the positive mindset that sets you up for success later in life. Just positive access, so that we can share with the world our strengths to the greater community." (caregiver) |

**Abbreviation:** MRI, magnetic resonance image

**Comments About Early Intervention and Therapy Access During Focus Groups**

| |
|---|
| Group 1: "I feel like that really helped and shaped my daughter to be in a better position than if she wouldn't have had that. So I honestly am a firm believer on the ECI program. That shaped my kid." (caregiver) |
| Group 1: "We were in ECI but we were driving an hour and a half, three days a week. But we were in ECI because we fight to get a speech therapist to drive. But she was supposed to do OT and PT, everything with us, and feeding." (caregiver) |
| Group 1: "As she's getting older, there's also less resources as they get older. Like, that's just personally what I've experienced. I mean, she's gonna be 14 and for two years, she hasn't had OT." (caregiver) |
| Group 1: "A lot of the things that they do is in North Dallas, like McKinney, Allen, like they're all in that area. And so that's another complaint that I have is there's not that many resources south of the DFW area." (individual with CP) |
| Group 2: "I put on mine the 'wait and see approach' or the waiting on a diagnosis. I think it goes into that because I have a kid who I think could have a diagnosis of cerebral palsy based on his presentation, his medical history, but they haven't given it to him yet. He's two and a half. So that's kind of where the disconnect comes in, and as a PT, I can't do medical diagnosis. And not that the diagnosis is everything, you know I treat what I see, but it is helpful." (clinician) |
| Group 2: "I don't understand what the doctor's waiting for, like 'come back in six months'. In that six months, so much could have happened therapeutically that could have helped." (clinician) |
| Group 4: "When we got out of ECI, I felt like we were thrown to the wolves, and so that's when I had to really start to push things. Because my insurance only covered 50 therapies a year, for my daughter. She needs more than that. So she needs PT, OT, and speech, and we weren't |

| |
|---|
| gonna get any of that because our insurance wouldn't cover it. So I had to go..I met with a Congressman, I visited a State Representative, I emailed the President, I did all the things looking to find a way to get my daughter what she needed." (caregiver/researcher) |
| Group 4: "We're not doing therapy because we can't really afford it right now. We both have good jobs, but still it's a LOT of money, even with insurance, to pay for therapy that she needs." (caregiver) |
| Group 7: "Stop 'the wait and see' mentality. When the pediatricians say, oh, let's wait and see, he just three months, let's wait, because we are losing precious time." (caregiver) |
| Group 8: "And it's never too early to present communication options, and it's never too early to present the opportunity to explore one's own environment. I've seen a child as young as 6 months in a power wheelchair to be able to explore their own environment." (clinician) |
| Group 8: "I like the fact that my mom had resources. Well, now it's called Easter Seals North Texas, where intensive therapy like Montessori classes, and PT, OT at a very early age. It was like 3 hours a day, 5 days a week and repetitive, so that was hopeful in early childhood before kindergarten." (individual with CP) |

**Abbreviations:** DFW, Dallas–Fort Worth; ECI, early childhood intervention; OT, occupational therapy; PT, physical therapy.

**Comments About Infrastructure and Accessibility During Focus Groups**

| |
|---|
| Group 1: "And like our school doesn't have a PT now." "Our area doesn't. I have to go in clinic now because we only have home health PTs." "That's the biggest problem I think. My daughter hasn't had OT in almost two years because of a shortage of therapists." (caregiver) |
| Group 1: "More support for rural areas." (caregiver) |
| Group 4: "I think beginning with schools, something I see is there's still some seclusion. I have some kiddos in my schools that come to my classroom. I'm like they could stay in there all day long, like they could, I mean, I don't know. I wish there was just more inclusion…" (caregiver/educator) |
| Group 4: "We got on the HCS (something) program, and because of getting on it – that's another thing – like my daughter got on it at 5, but the wait is 18 to 20 years, so if she did not have that, what would we be doing?"(caregiver) |
| Group 4: "I also put qualifications. I guess I'm kind of being a little stubborn in this…we have a placard for my daughter because she's got a wheelchair, but it's like everybody has a placard…" Interjection: "OH MY GODDD." "I know, and with qualifications too, it's like, will everybody get approved for a DAS pass now, and stuff? So it's just, I don't know, qualifications. I guess I'm being like, you don't get it because you're not disabled enough, and that's wrong." (caregiver) |
| Group 4: "And more advocates. I feel like we should have more advocates that help us through the transition from ECI to school because we didn't know what the heck we were doing." (caregiver) |
| Group 4: "And I just wanna put, just to stop having resources in one particular area. I feel like we should have resources everywhere, like the suburbs, the rural areas." (caregiver) |
| Group 4: "But yes that, and Morgan's Wonderland, everything is accessible. Everything. And it's not that hard. When you go there, you realize wow, the world really could be accessible; they just choose not to be because it's not cost effective." (caregiver) |
| Group 4: "Travel for kids with wheelchairs. I feel like that is a big headache when we're trying to take her chair on the plane. I can't take her normal wheelchair because it's got all these batteries and stuff, so then I went and purchased a smaller wheelchair that I can travel with. |

| |
|---|
| And even because that had a lithium battery, I went through the wringer. And then they just throw the wheelchair, and they always mess up something, like they did that with her stroller. And then if you if you take her wheelchair…we went to New York City. We got stuck in the subway at the bottom, and we had to carry her wheelchair up.” (caregiver) |
| Group 8: “I’ve heard it a lot ‘Oh we understand what they’re saying’. No, I want them to be able to communicate with the world at large, not just within the family and the teacher that understands them or their para that understands them, but the world at large.” (caregiver) |
| Group 8: “I hope that our legislature will improve funding for services in the community. Touching on what my ladies said here, it is a real struggle, both in ECI and IDD services. The programs are underfunded.” (clinician/researcher) |
| Group 8: “One thing I hope for is better access to medical services, like it would be my dream when I go to the primary doctor, that instead of asking me how much I weigh, that they would provide a scale that you could roll up.” (individual with CP) |
| Group 8: “And I know this is adult, but a well-known hospital turned her away because she said her wheelchair was not accessible to the equipment that they use to detect breast cancer. And like when they say ‘Hop on the table’ [laughing] And a lot of times, the buildings they say it’s accessible, but it’s not necessarily accessible in a way that makes it easier. Yeah, and I’m just wondering ‘What medical tests are we missing?’ Like as far as pap smears and all that in adulthood that you have to do, but how is that feasible for us to do that? (individual with CP) |
| Group 8: “I would say that perhaps there’s a space for a meetup like today, where you have whole families even, and parents could convene and fellowship even and then the kids, or the younger generation of the parents, are having fellowship and could also do something similar. It’s hard to get out of the house after school, in the evening, after work, but if it was all kind of in one place, but you still had your peers…” (individual with CP) |
| Group 8: “I notice that a lot of our communities, especially some of the communities that are developing programs and parks and things like that, and then even in schools, that there’s a lack of equipment and things like that in public parks for people that have disabilities. I notice that for young children—I taught at a school where we didn’t have a playground that was accessible for our children in wheelchairs. I think that’s something that indicates that we have a lack of awareness in the communities where people live.” (educator) |

**Abbreviations:** ECI, early childhood intervention; HCS, Home and Community-based Services; IDD, intellectual and developmental disabilities; OT, occupational therapy; PT, physical therapist/therapy.
