# Supplementary material for: Multi-Collaborator Engagement to Identify Research Priorities for Early Intervention in Cerebral Palsy
Source: J Clin Med. 2025 Oct 26;14(21):7592. doi: 10.3390/jcm14217592 (PMC12610828; doi:10.3390/jcm14217592)
Supplement: Supplementary file 1 [file jcm-14-07592-s001.zip › Supplementary Material S5.pdf]

Multi-Collaborator Engagement to Identify Research Priorities for Early Intervention in Cerebral Palsy

THE CASE OF THE MISSING RESEARCH PRIORITIES FOR YOUNG CHILDREN WITH CEREBRAL PALSY

Introduction

Early detection guidelines for cerebral palsy (CP) exist, but they are not consistently put into practice. Many children still experience delays in diagnosis and access to therapies during critical developmental years.

Identified major barriers include:

- Providers are not consistently trained in early detection tools.
- Systems lack time, staffing, and funding to support best practices.
- Policies and workflows make it difficult to coordinate referrals and interventions.

While these challenges have been reported globally, it was unclear what specific barriers existed in the southwestern United States—and how research could meaningfully improve early diagnosis and intervention for young children with CP in this region.

Objectives

To address this gap, the **Cerebral Palsy Task Force** was formed to engage families, adults with CP, clinicians, and researchers in setting research priorities. During this process, we discovered that “standard care” varies widely. Instead of launching comparative effectiveness research immediately, we first needed to:

Understand current care practices

Identify local barriers

Listen to family and clinician priorities

Build a roadmap to guide future research

This lay summary presents the shared research priorities and framework that emerged from this foundational work.

Who Participated?

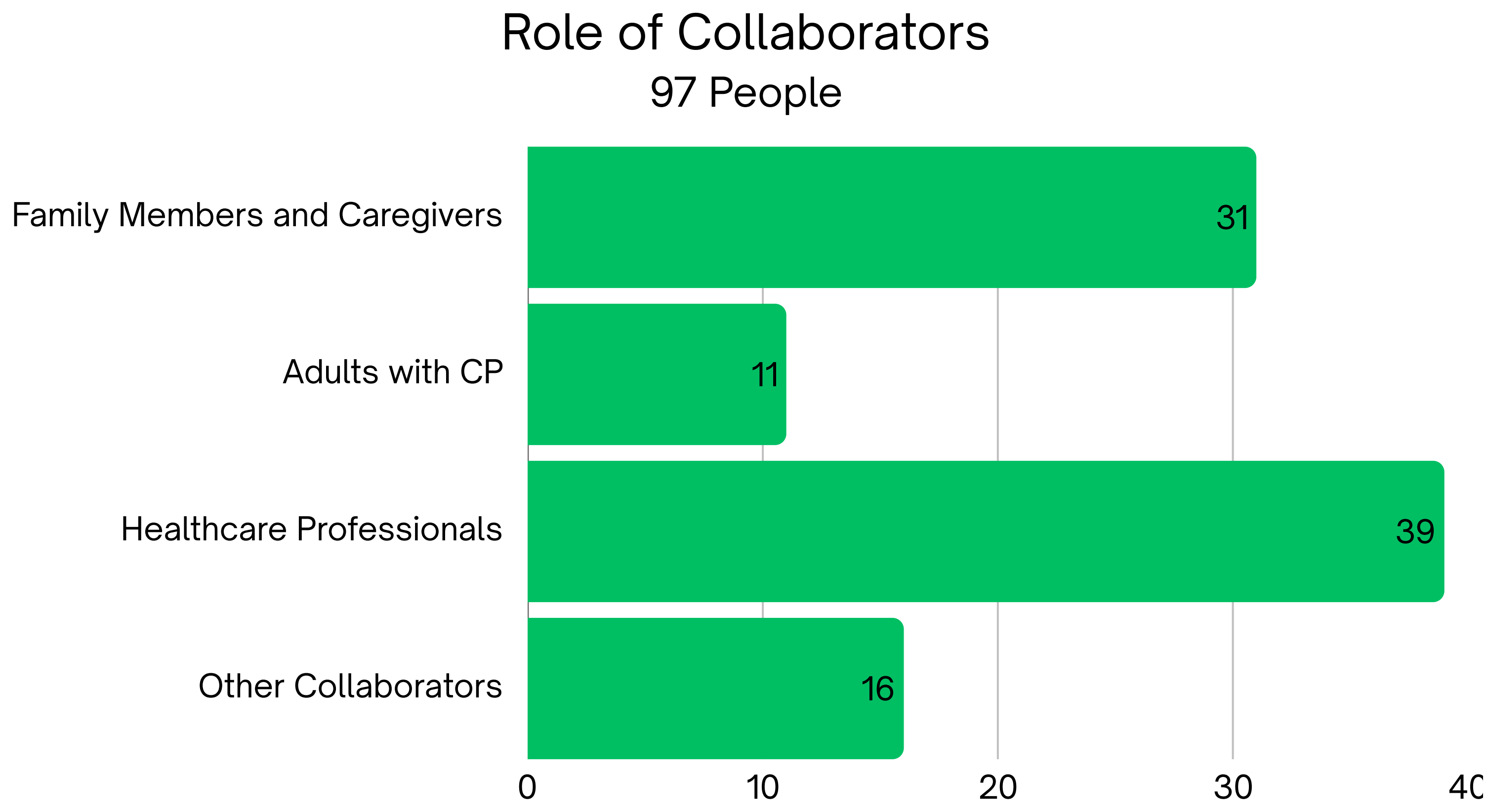

Process

January	A Task Force specific to setting research priorities for young children with CP was formed.
January-August	The Task Force met monthly to learn about patient centered comparative clinical effectiveness research and plan for the conference.
August-September	The pre-conference survey was distributed and completed.
September	The Task Force hosted a one-day hybrid (virtual and in-person) conference to identify research priorities for young children with CP and their families.
October	The Task Force reviewed focus group and survey data to identify actionable research items.
November	A follow-up survey validated the findings, and the Task Force collaboratively created a framework to address the research priorities.

Priority Themes

Theme 1 – Healthcare System Barriers
Inconsistent referrals, limited resources, and fragmented care systems.

Theme 2 – Lack of Awareness & Education
Families and providers lack access to CP-specific information and training.

Theme 3 – Communication & Compassion
Families need clearer, more empathetic conversations about diagnosis and care.

Theme 4 – Early Intervention & Therapy Access
Timely, engaging, and interdisciplinary therapy is not equally available.

Theme 5 – Infrastructure & Accessibility
Policies, community access, and service systems need improvement.

Actionable Framework

Improving Diagnosis Communication
→ Train providers to deliver information clearly and compassionately

Ensuring Early Referrals & Collaboration
→ Build coordinated networks and referral pathways

Expanding Education & Training
→ Strengthen provider skills and empower families to self-advocate

Scaling and Innovating Therapies
→ Grow access to proven interventions and test new strategies

Building Social Support Systems
→ Parent navigators, peer support, and advocacy networks

Driving Policy & Cultural Change
→ Reform insurance, promote inclusion, and raise public awareness

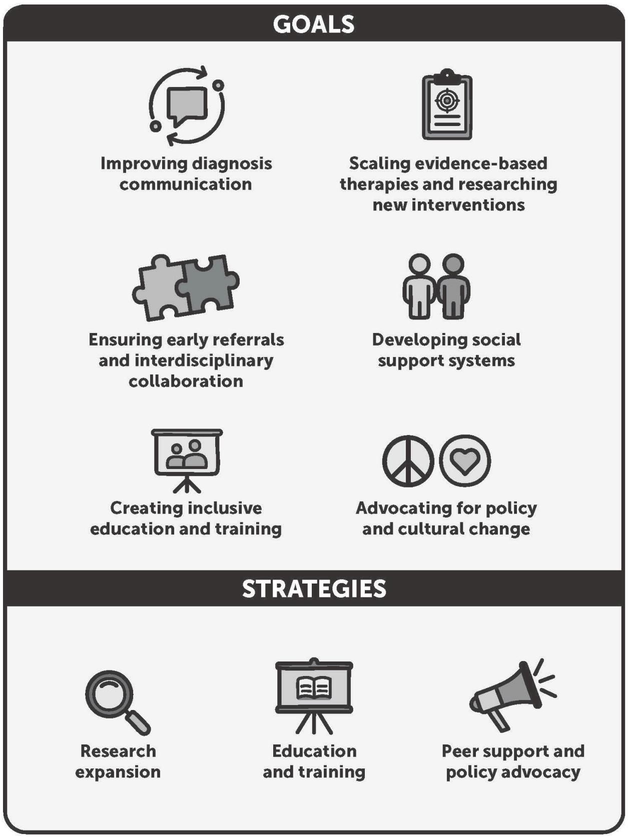

Why it Matters

These priorities will lead to earlier diagnosis, better care coordination, and more meaningful outcomes for children and families.

What Comes Next

Education & Training
- Expand CP training in schools and healthcare systems
- Share cost-effective and scalable strategies across regions

Research Expansion
- Test real-world implementation strategies
- Advocate for larger funding models

Peer Support & Advocacy
- Improve access to family supports and inclusive policies
- Address equity across socioeconomic backgrounds

Acknowledgements

Developed in collaboration with families, adults with CP, clinicians, researchers, and community partners.

Funding:
Supported by a Patient-Centered Outcomes Research Institute® (PCORI®) Eugene Washington Engagement Award (EASCS-32704).

The views expressed are those of the authors and do not necessarily reflect those of PCORI®. The funder had no role in data collection, interpretation, or reporting.

Shierk, A., Clegg, N. J., Fulton, D., Delgado, M. R., Hunt, V., Bettger, J., Chapa, S. M., Oakley, S. J., & Roberts, H. M. (in review). *Multi-collaborator engagement to identify research priorities for early intervention in cerebral palsy*. *Journal of Clinical Medicine*.

TEXAS WOMAN'S UNIVERSITY

SCOTTISH RITE FOR CHILDREN

UTSouthwestern Medical Center
